# The Natural Selection of Private and Inner Speech

**DOI:** 10.3389/fpsyg.2020.00163

**Published:** 2020-02-18

**Authors:** Sean O’Connor

**Affiliations:** Independent Researcher, Whittier, NC, United States

**Keywords:** evolution, natural selection, private speech, inner speech, evolutionary psychology, thought, language

## Abstract

This article analyzes the emergence of private and inner speech from the perspective of natural selection, arguing that social speech acts as a selection pressure for the emergence of private speech, that private speech acts as a selection pressure that leads to the emergence of inner speech, and that this view of private and inner speech may help to explain the natural selection of a variety of other traits of the human mind in an asymmetric intraspecific evolutionary arms race.

## Introduction

The natural selection of language is a complex and controversial topic that has been analyzed by many scientists and philosophers (Fitch, 2005) since Charles Darwin first applied the theory of evolution to attempt to explain human behavior (Darwin, 1871). However, understanding the natural selection of private and inner speech may be a key aspect of this investigation which has been overlooked in this debate. This is likely because private and inner speech in their present form depend on language; thus, it seems logical to assume that private and inner speech can only emerge in a species that is capable of language. However, this may be a logical fallacy, as I will argue that understanding the natural selection of private and inner speech may help to shine light on the evolution of and help shape our understanding of language and a variety of other traits of the human mind. To explain this view, I present the reader with a brief overview of the concept of natural selection, discuss the selection pressures that may be responsible for the propagation of private and inner speech, and explain how inner speech itself may have acted as a selection pressure which helps to explain the emergence of a variety of other traits in an asymmetric intraspecific evolutionary arms race.

## Natural Selection of Private and Inner Speech

According to Darwin (1859), evolutionary adaptation is primarily driven by natural selection. Natural selection is based on the idea that random mutations may increase an individual’s ability to survive and reproduce relative to other members of that species; thus, over time, such a random mutation may propagate, or spread, to an entire population. The ability of a random mutation to propagate in a species depends on the selection pressures that particular species faces. A selection pressure is a factor that affects the ability of the individual within a species to survive and reproduce. Common examples of selection pressures include predators, climate, disease, and competition for resources both within a species and between species. A common example used to understand how random mutations and selection pressures work together to drive the evolutionary adaptation of a species is how giraffes acquired such long necks. The theory is that over time individual giraffes acquired random mutations that made their necks longer and because a giraffe with a longer neck could reach food that both other giraffes and other species could not reach and thus increase their ability to survive and procreate, this trait was able to propagate through the species and eventually all giraffes had longer necks (Cameron and du Toit, 2007). In this example, the selection pressure that drove the natural selection of longer necks is both intraspecific and interspecific competition for food. The primary goals of this essay are to discuss what selection pressures would drive the natural selection of random mutations which allowed private and inner speech, and to argue that inner speech itself could create a selection pressure for the emergence of a variety of other traits in an asymmetric intraspecific evolutionary arms race.

Private speech is audible speech directed at oneself. The origin of the study of private speech is most often associated with Lev Vygotsky, who argued private speech is when “the social/cultural tool or symbol system of language, first used for interpersonal communication is used by the child overtly not for communication with others, but for intrapersonal communication and self-guidance” (Vygotsky, 1978). Vygotsky proposed that speech, in terms of development, transforms from interpersonal social speech, to intrapersonal audible “private speech,” to intrapersonal silent “inner speech” (Alderson-Day and Fernyhough, 2015). The functional purpose of private speech, to Vygotsky, was regulating one’s own cognition and behavior, with private speech being proposed to allow children to problem solve with themselves in order to perform more difficult tasks. Recent empirical research has been “largely supportive of Vygotskian claims about the functional significance of private speech, particularly its relations to task difficulty and task performance, and it’s developmental trajectory” (Alderson-Day and Fernyhough, 2015); however, private speech is also proposed to serve a variety of functions, such as pretense, practice for social encounters, language practice, etc. (Alderson-Day and Fernyhough, 2015).

While, to my knowledge, Vygotsky did not comment on the natural selection of private and inner speech, I will argue that his developmental view of private and inner speech can be translated similarly in terms of natural selection; in other words, that social speech acted as a selection pressure for the emergence of private speech, which acted as a selection pressure that led to the emergence of inner speech. When I write that trait A acted as a selection pressure for the emergence of trait B (a recurring phrase in this paper), I mean the emergence of trait A gives an evolutionary fitness advantage to an individual who possesses trait B, making trait B more likely to propagate.

For example, if Vygotsky was correct that private speech is social speech directed at oneself, from an evolutionary perspective private speech would not likely propagate in a species until social speech had reached a certain level of utility. This is because if an individual could not use social speech for problem solving, regulating the behavior and emotions of others, or teaching others, etc., the individual could presumably not use private speech to problem solve with themselves, regulate the behavior and emotions of themselves, or teach themselves, etc, since the individual is speaking to themselves as they would speak to another. Thus, an individual who could not use social speech for problem solving with others, regulating the behavior and emotions of others, etc., who had a random mutation that allowed private speech would be no better off from an evolutionary fitness standpoint and private speech would not likely propagate. In this sense, social speech itself may have acted as a selection pressure for the emergence of private speech, as the more advanced social speech is, the more evolutionarily beneficial a random mutation that allowed private speech would be.

If inner speech is private speech internalized, as Vygotsky suggested, this begs the question; what selection pressure would allow the propagation of a mutation that allowed private speech to become internalized? The selection pressure I will argue accomplished this evolutionary feat is the need to hide one’s private speech from others; in other words, to hide what one was planning, to hide how one prepared to solve a problem, to hide how one was regulating their own behavior and emotions, or to hide what one learned, etc. In a social situation an individual would need to hide their private speech from others if their use of private speech would get them in trouble, thus, the need to hide one’s private speech from another would be driven by selfish private speech. This use of selfish is from an evolutionary perspective, meaning the use of private speech to increase an individual’s own fitness at the expense of the fitness of the community. A simple example used to demonstrate this is that if an individual in a group spots food that is inaccessible and makes a plan via private speech to obtain that food, the group may detect the individual’s plan and can use that plan to get the food themselves; yet if the individual makes this plan via inner speech, the group will be unaware of the individual’s plan and the individual can wait until they are alone to retrieve the food, raising the evolutionary fitness of the individual at the expense of the community. As this inner speech was hidden from others and yet could change the behavior of an individual, I may refer to this usage of inner speech as “motives” which can be defined as “a reason for doing something, especially one that is hidden or not obvious” (Motive, 2019).

Cheating social cooperation with private speech, however, would not likely lead directly to the emergence of inner speech – rather, I will argue that cheating social cooperation with private speech would act as a selection pressure for the detection and punishment of cheating with private speech, which would act as a selection pressure for traits that allowed an individual to hide cheating with private speech from others. Inner speech is argued to be a result of this selection pressure – the need to hide selfish private speech. This form of evolution is known as an asymmetric intraspecific evolutionary arms race, which occurs when competing selection pressures drive the propagation of traits within a single species, contrasted with a symmetric interspecific evolution arms race, which occurs when a single selection pressure drives the propagation of traits between two species – such as two species of trees growing taller due to the same selection pressure of the need for sunlight. The two competing selection pressures in this asymmetric intraspecific evolutionary arms race are proposed to be the need to take advantage of and maintain social cooperation. For example, cheating with private speech takes advantage of social cooperation, whereas detecting and punishing cheating with private speech maintains social cooperation by preventing cheating with private speech, and inner speech again takes advantage of social cooperation by facilitating cheating with private speech internally.

This is a tempting way to view the evolution of inner speech because this follows a pattern of evolution that we can see in other species. For example, I have argued cheating traits may create a selection pressure for traits that allow cheating-detection and the punishment of cheaters to prevent cheating and raise the fitness of the cheated. An example of this in the animal world can be seen in the behavior of Galada monkeys (le Roux et al., 2013). As a form of social cooperation female Galada monkeys typically only mate with the dominant leader male, however, females and non-leader males can cheat this social cooperation by mating with each other. According to the pattern argued above, this cheating should act as a selection pressure for the emergence of traits that allow the detection and punishment of cheating – and indeed, if a leader male discovers a female and another male mating, the leader male aggressively chases the cheaters apart from one another. This detection of cheating occurs if the leader male either sees the cheating, or hears the cheating, as female and male Galada monkeys typically give loud calls while mating. Furthermore, according to the pattern argued above, this detection and punishment of cheating should act as a selection pressure for the emergence of traits that allow hiding from the detection and punishment of cheating – and indeed, after observing sexual relationships among Galada monkeys, researchers determined that cheating individuals make sexual noises less frequently and tend to mate only when the leader male was at a far enough distance to not hear or see the mating occur (le Roux et al., 2013). This is an example of how the detection and punishment of cheating social cooperation may create a selection pressure for the emergence of traits that allow the hiding of cheating, just as the detection and punishment of cheating with private speech has been proposed as a selection pressure for the emergence of inner speech.

This view of inner speech as the result of the need to hide selfish private speech would not likely be the last step in this arms race. As a trait that allows an individual to cheat social cooperation, inner speech may act as a selection pressure for the emergence of the detection and punishment of the selfish inner speech of others, just as cheating with private speech has been proposed as a selection pressure for the emergence of the detection and punishment of selfish private speech. Furthermore, the detection and punishment of cheating with inner speech would act as a selection pressure for the ability to hide from the detection and punishment of cheating with inner speech, just as the detection and punishment of selfish private speech was proposed as a selection pressure for inner speech. If we can consider inner speech motives, we can translate this as selfish motives acted as a selection pressure for the detection of the selfish motives of others, and hiding one’s own selfish motives from others. As inner speech is inherently undetectable, the detection of selfish motives in my opinion may best be understood as traits which allowed reading body language – facial expression, eye gaze, anxiety level, emotions, etc., and hiding from the detection of selfish motives may be best understood as using deceptive body language. Finally, as Hippel and Trivers have argued that self-deception facilitates deception (Hippel and Trivers, 2011), I would argue that hiding one’s own motives from oneself – unconscious motives – may have also emerged as a trait that helps to hide one’s own motives from others.

Thus far I have argued that social speech created a selection pressure for the emergence of private speech, that cheating with private speech created a selection pressure for the emergence of the detection and punishment of cheating with private speech, and that the detection and punishment of cheating with private speech created a selection pressure for the emergence of hiding cheating with private speech: inner speech. Furthermore, I have argued that this pattern is repeated, as cheating with inner speech created a selection pressure for the emergence of the detection and punishment of cheating with inner speech and the detection and punishment of cheating with inner speech created a selection pressure for hiding signs of inner speech from both others and oneself. This information is summarized in Table 1. In the next section I will explain how this pattern may have been repeated one more time due to the emergence of speech about inner speech.

**TABLE 1:** This table summarizes how private and inner speech are proposed to have emerged in an intraspecific evolutionary arms race whereby each emerged trait acts as a selection pressure for the emergence of each subsequent trait.

Selection pressure	Emerged trait
Social speech	Private speech
Cheating with private speech	Detection and punishment of cheating with private speech
Detection and punishment of cheating with private speech	Hiding cheating with private speech via inner speech
Cheating with inner speech	Detection and punishment of cheating with inner speech
Detection and punishment of cheating with inner speech	Hiding cheating with inner speech from others and oneself

## Speaking About Inner Speech

In addition to the traits mentioned above, this view of the evolutionary progression of private and inner speech may help to explain the emergence of the ability to communicate inner speech to others, possibility providing a mechanism for, more generally and colloquially termed, the ability of an individual to speak about what they are thinking. For example, if family A has been captured by family B, an individual in family A who has used inner speech to make a plan to escape may be much better off if they can share their plan to escape with other members of family A, both to assist in the escape and to help the individual from group A to survive after escaping family B. Thus, inner speech may create a selection pressure for the emergence of speaking about inner speech.

Importantly, this speech about inner speech would act as a form of social cooperation, and therefore present an opportunity for the emergence of traits that allowed an individual to cheat this social cooperation, causing speech about inner speech to follow the same pattern that private and inner speech have been proposed to follow; cheating, detection/punishment of cheating, and hiding from the detection/punishment of cheating. For example, a trait that may allow an individual to cheat by speaking about their inner speech is the internal rehearsal of that speech – inner speech about inner speech – more generally and colloquially, thoughts about thoughts. By internally rehearsing this speech about inner speech to themselves, an individual may learn how their own speech-about-inner speech would be viewed by another as a form of “practice for social encounters,” which has been proposed as a function of inner speech (Alderson-Day and Fernyhough, 2015). In a complex social society, understanding the effects an individual’s own speech has on others could increase an individual’s evolutionary fitness, for example, by preventing the individual from saying things which would arouse negative reactions in others. Furthermore, this trait may act as a “cheating” trait by facilitating the manipulation of others via deceptive speech, or “lying.” For example – if inner speech about inner speech allows individual A to understand that speaking a lie to individual B will cause individual B to drop their food and run away, individual A will have the opportunity to grab that food and increase their own chances of survival at the expense of individual B.

As with cheating with private and inner speech, the ability to cheat with speech about inner speech would create a selection pressure for the detection and punishment of cheating with speech about inner speech, or the detection and punishment of lies, such as understanding when another lied about their motives for committing a crime. Again more generally and colloquially speaking, this may be considered thinking about the thoughts of others. Furthermore, similar to how inner speech was argued to create a selection pressure for the emergence of speaking about inner speech, thinking about the thoughts of others may create a selection pressure for the emergence of speaking about the thoughts of others; for example, to continue the example above, if family A was captured by family B and an individual of family B determined that an individual in family A was planning to fight family B to escape, the individual from family B would be more likely to survive such an attack if they could tell other members of family B what they thought the individual of family A was planning.

Furthermore, as argued earlier about detecting and punishing cheating with private and inner speech, detecting and punishing cheating with speech about inner speech would create a selection pressure for traits that would allow an individual to hide signs of deceptive speech about inner speech from others and themselves, such as denial, when an individual pretends to others and themselves that they did not use deceptive speech about inner speech – that they were not lying.

To extend this model of cheating > detection/punishment > hiding from detection beyond that which we have seen in nature, I would argue that denial may then create a selection pressure for information seeking behavior; in other words, if individual A lies about their motives for committing a crime, the community will be better off if they can use information seeking behavior to determine the truth; for example, asking the accused what happened, where it happened, when it happened, etc. This is consistent with the work of Spink and Cole, who have proposed that human information seeking behavior is the result of an intraspecific evolutionary arms race (Spink and Cole, 2007). Finally, the emergence of information seeking behavior is important because information seeking behavior may act as a selection pressure for an individual to be able to give information, or speak about their internal and external experience – what they feel, what they hear, what they saw, when they saw it, where they saw it, etc. As I have argued that an individual can speak about their inner speech, I will conclude by speculating that the need to speak about their internal and external experiences may be solved by conducting inner speech about these internal and external experiences and speaking about this inner speech.

In this section, I heard argued that the view of inner speech as private speech internalized may create a selection pressure for the emergence of the ability to speak and lie about the inner speech of others and themselves to others and themselves internally, detect, punish, and avoid the detection of cheating with speech about inner speech, seek information, and give information – or speak about the internal and external experiences that caused them to behave a particular way. This is summarized in Table 2. Due to these reasons, I believe a better understanding of the natural selection of private and inner speech and the traits that may have arisen due to inner speech may help to explain the emergence and shape our understanding of human language and the human mind.

**TABLE 2:** This table summarizes how speech about inner speech is proposed to have emerged and follow a similar evolutionary pattern as has been argued for private and inner speech, whereby each emerged trait acts as a selection pressure for the emergence of each subsequent trait.

Selection pressure	Emerged trait
Inner Speech	Speech about Inner Speech
Cheating with speech about inner speech via inner speech about inner speech	Detection and punishment of cheating with speech about inner speech
Detection and punishment of cheating with speech about inner speech	Hiding cheating with speech about inner speech from others and oneself
Hiding cheating with speech about inner speech from others	Information seeking behavior
Information seeking behavior	Information giving behavior

## Conclusion

In this essay, I have argued that advanced interpersonal speech creates a selection pressure for the emergence of private speech, which creates a selection pressure for the emergence of inner speech due to a selfish evolutionary need to avoid the detection and punishment of cheating with private speech. Furthermore, I argued that this view of inner speech may help to explain the emergence of a variety of traits in an asymmetric intraspecific evolutionary arms, including the ability of an individual to speak to others and themselves about the inner speech of both others and themselves, lie about the inner speech of themselves to others and themselves, detect and punish deceptive inner speech and deceptive speech about inner speech, and avoid the detection and punishment of deceptive inner speech and deceptive speech about inner speech. Finally, I argued that avoiding the detection and punishment of deceptive speech about inner speech created a selection pressure for the emergence of information seeking behavior, which created a selection pressure for the emergence of the ability to give information, or speak about one’s own internal and external experience.

To further summarize, this theory has proposed an evolutionary reason for and relative order of the emergence of certain traits of the human brain. As such, it will be difficult to support experimentally; however, there is hope that within the next few decades DNA analysis of fossils may be used to determine the “evolutionary timing of novel human capacities including neural circuitry” (Fitch, 2018). Such analysis could provide experimental support for the relative order of the emergence of traits that has been proposed in this article. While correlation does not necessarily mean causation, such a correlation would provide support for the evolutionary reason for the emergence of each trait – in other words, that each trait acted as a selection pressure for the emergence of the subsequently evolved trait.

Thus, while this theory has been written from a largely speculative evolutionary perspective, I believe this is a novel view of the natural selection of private and inner speech that could help to further the scientific understanding of the evolution of the human mind and language, and deserves both the attention of evolutionary theorists and attempts at integration with other areas of science such as psychology, genetics, sociology, and cognitive neuroscience.

## Author Contributions

SO’C was the sole contributor to the manuscript.

## Conflict of Interest

The author declares that the research was conducted in the absence of any commercial or financial relationships that could be construed as a potential conflict of interest.
